# The Design of a Sustainable Industrial Wastewater Treatment System and The Generation of Biohydrogen from *E. crassipes*

**DOI:** 10.3390/polym16070893

**Published:** 2024-03-25

**Authors:** Uriel Fernando Carreño Sayago

**Affiliations:** Facultad de Ingenieria, Fundacion Universitaria los Libertadores, 111221 Bogotá, Colombia; ufcarrenos@libertadores.edu.co

**Keywords:** *E. crassipes*, chromium (VI), biohydrogen, biomass

## Abstract

Water scarcity is a significant global issue caused by the prolonged disregard and unsustainable management of this essential resource by both public and private bodies. The dependence on fossil fuels further exacerbates society’s bleak environmental conditions. Therefore, it is crucial to explore alternative solutions to preserve our nation’s water resources properly and promote the production of biofuels. Research into the utilization of *E. crassipes* to remove heavy metals and generate biofuels is extensive. The combination of these two lines of inquiry presents an excellent opportunity to achieve sustainable development goals. This study aims to develop a sustainable wastewater treatment system and generate biohydrogen from dry, pulverized *E. crassipes* biomass. A treatment system was implemented to treat 1 L of industrial waste. The interconnected compartment system was built by utilizing recycled PET bottles to generate biohydrogen by reusing the feedstock for the treatment process. The production of biological hydrogen through dark fermentation, using biomass containing heavy metals as a biohydrogen source, was studied. Cr (VI) and Pb (II) levels had a low impact on hydrogen production. The uncontaminated biomass of *E. crassipes* displayed a significantly higher hydrogen yield (81.7 mL H_2_/g glucose). The presence of Cr (IV) in *E. crassipes* leads to a decrease in biohydrogen yield by 14%, and the presence of Pb (II) in *E. crassipes* leads to a decrease in biohydrogen yield of 26%. This work proposes a strategy that utilizes green technologies to recover and utilize contaminated water. Additionally, it enables the production of bioenergy with high efficiency, indirectly reducing greenhouse gases. This strategy aligns with international programs for the development of a circular economy.

## 1. Introduction

The dried and ground biomass of *E. crassipes* has been used successfully to remove heavy metals. The presence of hydroxyl (OH) and carboxyl (COOH) groups facilitates cation exchange with heavy metals. These groups were evidenced using FTIR tests [1,2,3,4,5,6]. Continuous treatment systems with fixed biomass have been used to purify water by exposing it to cellulose. This novel method yields reliable results and could be implemented in industries that contaminate water resources with their effluents [7,8,9,10]. Continuous systems are economical and easy to construct, as they are made with recycled materials [11]. Several studies have concluded that this biomass has effective chemisorption mechanisms for removing heavy metals [12,13,14]. In similar studies, the remotions of chromium (VI) was around 80% in tannery waters [14].

However, the final disposition of cellulose biomass loaded with metals remains unclear in various investigations. This can lead to potential environmental impacts as the biomass retains its molecular structure. To address this issue [15,16,17], researchers have utilized *E. crassipes* biomass after a phytoremediation process to produce bioethanol.

In other processes, the *E. crassipes* biomass has been used to successfully produce biohydrogen [18]. Biological production takes place in various bioreactors depending on the metabolic bioprocess and microorganism used, including dark fermentation [19,20,21,22,23,24,25,26,27,28].

Biohydrogen is defined as hydrogen produced biologically, usually by algae, bacteria, and archaea. It is a potential biofuel that can be obtained from crop waste and organic materials. Its use has increased globally due to its clean and highly efficient energy production [29,30,31,32,33,34,35,36].

Hydrogen-producing bacterial species belong to the genera *Enterobacter*, *Bacillus*, and *Clostridium* and are present in biosolids generated in water treatment plants (WTRs) [37,38,39,40]. The wastewater treatment plants in Salitre, Bogota, Colombia, have bacterial profiles dominated by the genera *Enterobacter*, *Bacillus*, and *Clostridium* [41,42,43].

These bacteria thrive in the anaerobic digestion processes that occur in the treatment plants, creating an ideal environment for their growth [44,45,46,47,48,49,50]. The fermentation by-products are acetic acid (with a theoretical maximum of 4 mol H_2_/mol glucose) and butyric acid (with a theoretical maximum of 3.4 mol H_2_/mol glucose). Therefore, practical yields of hydrogen from dark fermentation are around 2 mol H_2_/mol glucose [51].

The aim of this project is to merge two environmentally sustainable processes. Dried and crushed plant material from the *E. crassipes* plant will be used to eliminate Cr (VI) and Pb (II). Subsequently, this material will be used as a source for the production of hydrogen in dark fermentation bioreactors using biosolids from water treatment waste (WTR). The molecular power of this biomass for adsorbing heavy metals and producing biofuels, such as H_2_, will be determined.

## 2. Materials and Methods

Material vegetal. The plants were washed and dried and then cut into pieces, with the aim of obtaining particles of 0.212 mm in diameter.

Characterization of Eichhornia crassipes. Physicochemical characterization was carried out to identify the properties of the collected macrophytes, determining the structural carbohydrates and lignin content. In addition, the quantification of the biomass matrix used was carried out considering the following parameters: (a) % hemicellulose, (b) % cellulose, (c) % lignin, and (d) ash [39,40].

For the extraction of cellulose, an extract of benzene and ethanol with a volume ratio of 2:1 was used. For the extraction of hemicellulose, nitric acid and ethanol were used in a ratio of 1:4. For the extraction of lignin, 12% hydrochloric acid was used, and for the determination of ash, 72% hydrogen supplied was used, according to the National Standard Analysis of China (GB 2677).

Measurement of chromium and lead: Samples were taken in the flask at each time interval, analyzing the concentrations of chromium and residual lead. Then, 20 µm samples were taken and placed in the centrifuge (KASAI MIKRO 200). Upon sampling, aliquots of the reaction mixture were analyzed for residual chromium concentration using a UV84.

The 1000 (mg/L) Cr (VI) stock solution was prepared using potassium dichromate and distilled water. Similarly, a lead solution with a concentration of 1000 (mg/L) was prepared from lead nitrate. To maintain consistency with the experimental conditions, all samples were kept at a pH of 6.5.

The amount of chromium (VI) residue was estimated using the diphenylcarbazide method, which involves the determination of chromium and lead. To carry this out, 200 µL of 0.5% diphenylcarbazide (96% purity) was added to an Eppendorf tube, followed by 900 µL of phosphate buffer and 100 µL of the residual sample. The resulting mixture was then transferred to an absorption cell, and the absorbance was measured at 540 nm.

Based on the APHA (American Public Health Association Procedure) standard for standard tests (standard methods for the examination of wastewater), the tests for chromium and lead were performed.

**Figure 1:**
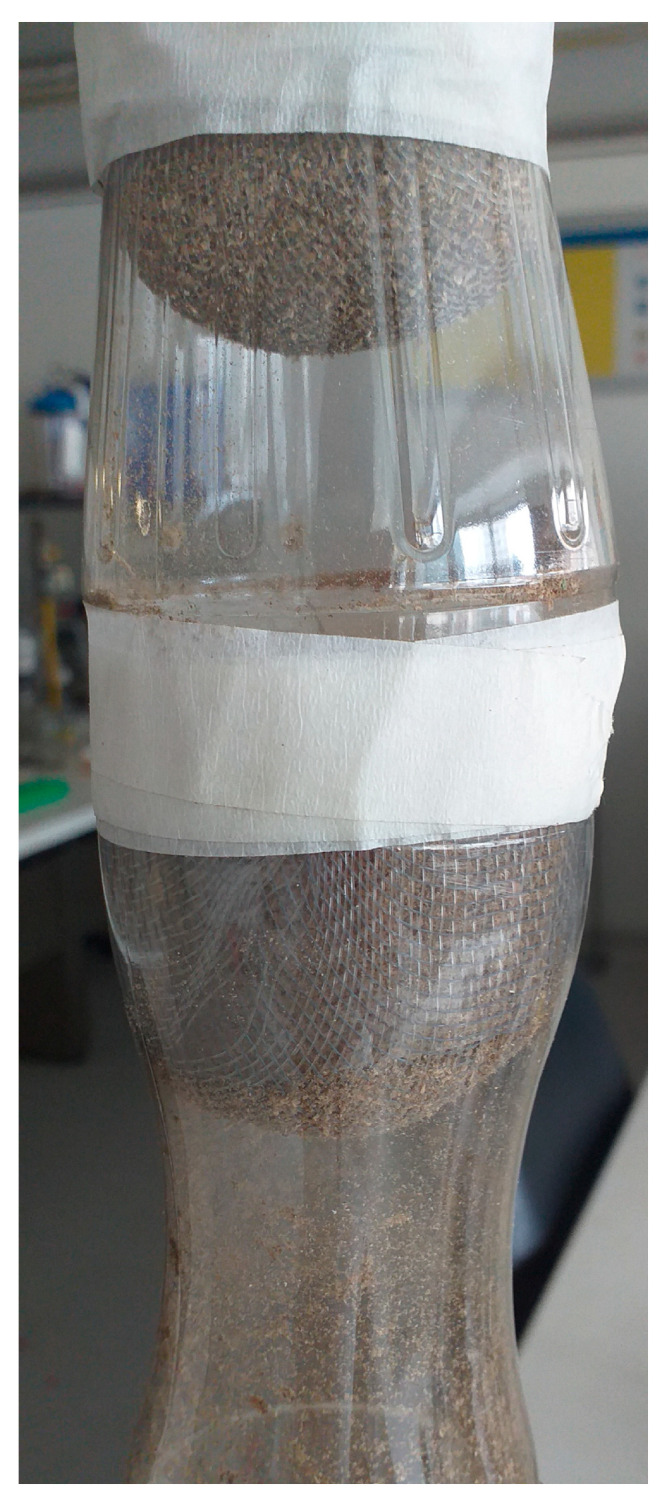
Bioremediation. Continuous experimentation.

During Phase 1, bioremediation was conducted through continuous experimentation using recycled 1 L bottles. The biomass was divided into two areas of 75 g each, as shown in Figure 1. To maintain a constant flow rate of 15 mL/min, the funnel effect of the bottles was utilized while keeping other parameters, such as pH (6.5) and temperature (18 °C), constant. The initial concentrations of chromium (VI) and lead (II) were 1000 mg/L. Each experiment was repeated three times, and the average value was used to generate the graphs in the results.

The Thomas model was used to determine the behavior of adsorption. Its parameters helped in determining breakthrough curves, adsorption capacities, and predicted first- and second-order kinetics. The model is correlated with the Langmuir isotherm. The Carreño model is used to determine the design parameters of this type of system [1,2,3,4,5], in the Table 1, is the Adsorption models.

We utilized the TESCAN FE-MEB LYRA3 scanning electron and focused ion beam microscope and EDS (energy dispersive X-ray spectroscopy).

Phase 2: Hydrogen production. The design of the biohydrogen generation process consists of two bioreactors: a bioreactor to produce the hydrolyzate and a bioreactor for dark fermentation.

Hydrolysis of *E. crassipes*: The hydrolyzate bioreactor was 5 L, as measured in the glass, had a lid for gas release, pH, and temperature sampling, and was placed in a heater with magnetic stirring at 120 rpm at a temperature of 30 °C.

For the hydrolysis process, three types of biomasses were taken, two used in the bioremediation process (lead treatment and chromium treatment) and another not used in this process.

In the bioreactor, 200 g of dried *E. crassipes* (from the three samples to be evaluated) were mixed with 500 mL of water. The samples underwent alkaline hydrolysis using 1% (*w*/*v*) NaOH at 45 °C for 12 h, with 400 mL of the reagent added. The samples were then washed with tap water to prevent negative chemical reactions in the next step.

Next, 3% (*v*/*v*) sulfuric acid (H_2_SO_4_) was added at 45 °C for 12 h, with 400 mL of the reagent added. The samples were then washed with tap water to remove the reagent and prevent reprocessing during dark fermentation.

The sugar content was determined using the dinitro salicylic acid (DNS) method. A four-liter solution of *Eichhornia crassipes* hydrolysate was obtained for biohydrogen production [14].

Biohydrogen was produced. The bioreactor for dark fermentation is designed with a 2 L glass vessel and a gas release lid. It is ideal for continuous sampling and was placed in a heater with magnetic stirring at 120 RPM and a temperature of 45 °C. The biosolids generated from the treatment of brackish water (WTR) from the city of Bogotá were used. The biosolids used in this study were produced through 20 days of anaerobic digestion. They were chosen for their bacterial profile, which has been characterized in previous studies. The bacterial species present belong to the genera *Enterobacter*, *Bacillus*, and *Clostridium* (WTR) [31,32,33].

Three bioreactor setups were used: two to evaluate biomass loaded with Cr (VI) and Pb (II) and one with biomass without heavy metals. 

As a result, the hydrolyzate procedure was carried out three additional times. Each bioreactor was filled with 100 g of hydrolysate, and 100 g of inoculum (biosolid) was added. The initial pH was 5.5. Rubber septa and aluminum plugs were used to hermetically seal the bioreactors, and the flask holes were sterilized with alcohol. The biogas volume was measured every 30 min using the plunger displacement method.

**Figure 2:**
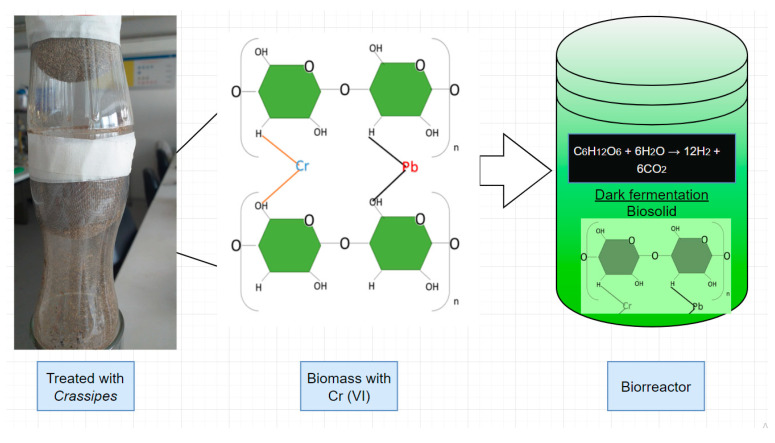
Bioremediation and biohydrogen sustainability production process.

Hydrogen gas was determined by gas chromatography using a TCD detector on a GC-Agilent 7890 chromatograph. The optimal temperature for hydrogen production is 47.5 °C [14]. In the Figure 2 is shows the bioremediation and biohydrogen sustainability production process.

The results of the different tests were determined with the Gompertz equation.

(3) $$H=Hmax*exp+\left(\left(-\exp\left(\frac{Rmax*exp}{Hmax}\right)\right)\right.\left(\alpha -t\right)+1)$$

where *α*: latency time; *R_m_*: maximum rate of H_2_ production; *H_max_*: maximum production potential.

## 3. Results

### 3.1. Results of Characterization Chemistry

*E. crassipes* collected in the wetland had a hemicellulose content of 33% and 30% cellulose, lignin was lower at 9% and ash content was high at 23%, due to the contamination inherent to the plant.

In different studies carried out, where the cellulose of *E. crassipes* has been physicochemically characterized, the high presence of cellulose and hemicellulose in its chemical composition has been demonstrated, as in the case of [39,40]. The presence of these two polysaccharides favors the process of metal adsorption and the production of biofuels [44,45,46,47]. The presence of lignin makes hydrolysis necessary before the biohydrogen production process [15,16,17].

### 3.2. Results of Adsorptions of Cr (VI) and Pb (II)

**Figure 3:**
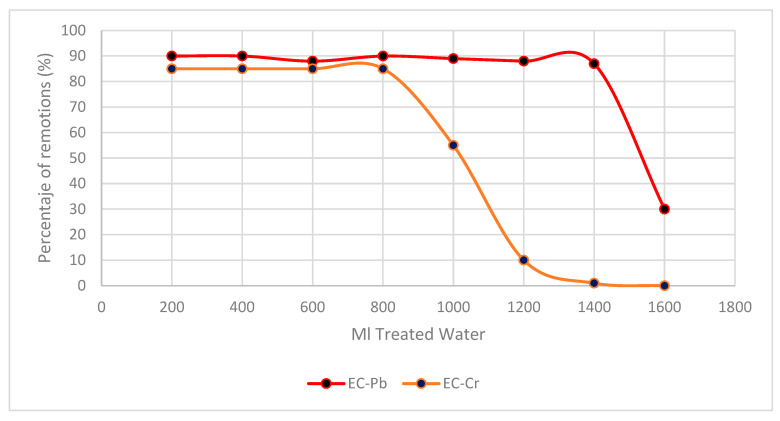
Removal of Cr (VI) and Pb (II) by biomass EC on percentages.

Figure 3 shows the different removal processes in EC biomass. A treatment with two different heavy metals, lead and chromium, was carried out. 

The Thomas model was adjusted to reflect the experimental results, which also follow the Langmuir isotherm. This led to the conclusion that the biomass’s superficial active sites are homogeneous [1]. As a result of this adjustment, we can use Model (1) for adsorption capacities. For comparison, we also used Carreño’s model (2) and found that the adsorption capacities for Cr (VI) and Pb (II) were 10 mg/g and 18 mg/g, respectively. Equations (1) and (2) relate the volume of water to the amount of biomass used.

The mechanism of adsorption occurs when heavy metals react with cellulose, forming chelating complexes and creating active sites. When Cr (VI) and Pb (II) pollutants come into contact with *E. crassipes* biomass, cationic exchanges occur. The pollutants are chemisorbed and exchanged. The presence of hydroxyl groups in the biomass facilitates these processes.

Previous research has demonstrated the efficacy of *E. crassipes* biomass in removing Cr (VI) and Pb (II) from continuous systems. For instance, in [1,2,3,4], over one liter of Cr (VI)-contaminated water was treated, resulting in the removal of approximately 80% of the chromium found in tannery wastewater, with around 50 g of this biomass. 

While chemical agents were not utilized in increasing the adsorption capacity or for the recycling processes, the successful Cr (VI) and Pb (II) adsorption processes treated over one liter of water contaminated with these heavy metals. The aim of avoiding chemical agents is to prevent cellulose alteration and subsequent negative effects on bio-hydrogen production, preserving the next level of sustainability.

### 3.3. Analysis EM y EDS

**Figure 4:**
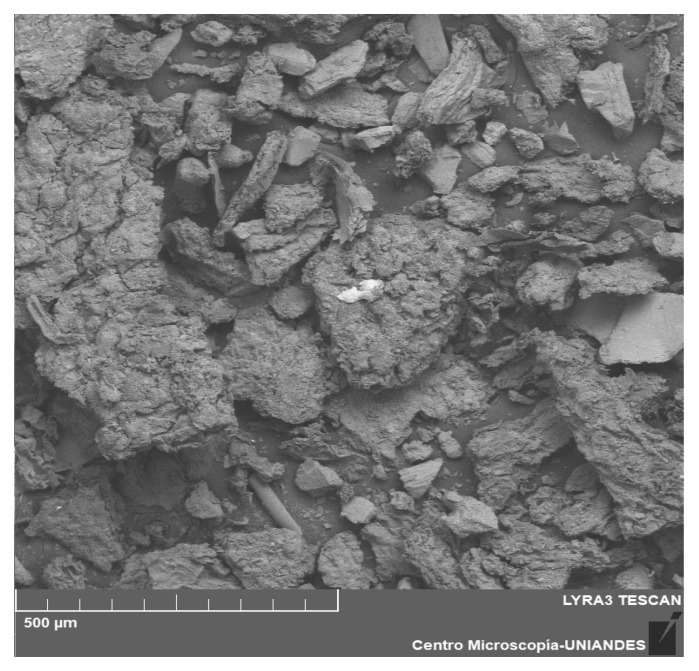
Characterization of without heavy metal.

Figure 4 shows the macro photography of biomass without heavy metal. 

**Figure 5:**
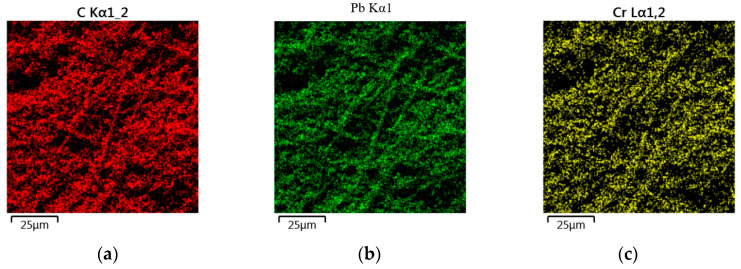
Characterization of biomass after adsorption of Pb (II) and Cr (VI) in the (**a**) carbon element, (**b**) Pb (II), and (**c**) Cr (VI).

Figure 5 shows the presence of carbon, which is fundamental in the structure of cellulose and hemicellulose, corroborating what is indicated in the physicochemical characterization carried out and shown in Table 2.

After Cr (VI) adsorption on the EC-Cr biomass, the surface morphology changed drastically, whereby the surface had a more irregular shape, showing an agglomerate-like surface and the presence of white ion particles. Cr (VI) on the surface confirms that the adsorption process occurred. The EDX spectrum showed that EC-Cr proves the adhesion of Cr (VI).

The observations of carbon and Cr (VI) in the EDX spectra of pearl show that the crosslinking processes have taken place efficiently between the polymeric components of the cellulose; these findings are summarized in Table 3, showing the characterization of the EC-Cr through EDS.

A percentage of 14.37% of Cr (VI) can be seen in the sample taken in dry weight on average of the three samples taken, representing a considerable amount adhered to the samples taken. Cr (VI) is chemisorbed due to cation exchange processes between hydroxyl (OH) groups and this heavy metal [3]. Subsequently, it will go through the hydrolysis process and subsequent production of biohydrogen.

After the adsorption of Pb (II) in the EC-Pb biomass, the presence of Pb (II) ion particles on the surface confirms that the adsorption process has occurred. The EDX spectrum showed that EC-Pb proves the adhesion of this heavy metal.

The observations of carbon, oxygen, and Pb (II) in the EDX spectra of the pearl show that the adsorption processes in the cellulose; these findings are summarized in Table 4.

It can be seen in Table 3 that, like the biomass loaded with Cr (VI), lead remains at a significant percentage of 16.6% in dry weight; this percentage is slightly higher compared to EC-Cr because with this metal, there was a better performance in water treatment, and it removed more; this could change the production of biohydrogen in the next stage.

### 3.4. Hydrolysis Results

The best performance in sugar production was the hydrolyzate without any metal attached, with a production of 180 g/L of sugars; the biomass with the biomass hydrolyzate EC-Pb obtained a production of around 120 g/L, and the biomass EC-Cr gave a result of 110 g/L. In other experiments, the hydrolyzate of *E. crassipes*, without heavy metals attached, always yielded satisfactory results [15].

### 3.5. Production of Biohidrogen

**Figure 6:**
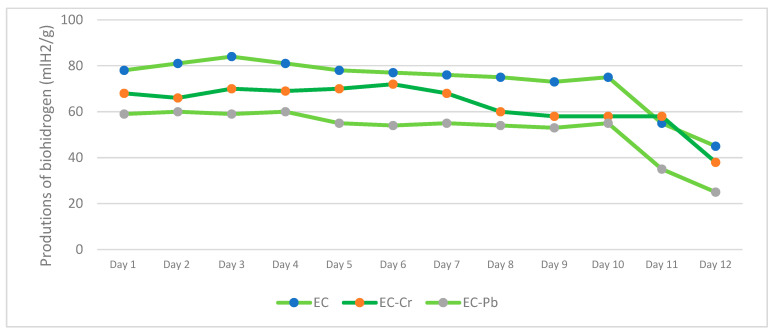
Production of biohydrogen in the three bioreactors.

Figure 6 shows the results of biohydrogen production for 10 continuous days of productivity.

The percentage of humidity in the three samples was around 90% because this type of dark fermentation process generates humidity in the samples [27]. The hydrogen gas yield remained constant over 12 days in all three experiments. However, in the biomass devoid of any heavy metal, it decreased to half its initial value once the inoculum was consumed; this is illustrated in Figure 6, where the yield drops from 80 to 40 mL H_2_/g glucose. On the third day, the specific hydrogen production rate peaked at 81.3 mL H_2_/g. 

During the EC-Cr biomass test, about 70 mL H_2_/g of biofuel was produced, which is significant despite a 10 mL decrease in comparison to H_2_ production by EC without heavy metals. In spite of the decrease, it can be inferred that Cr (VI) has no consequential impact on biohydrogen production, which is a vital contribution to ecological sustainability; these two processes can be combined in major projects.

Regarding the EC-Pb biomass, there was a reduction of 20 mL H_2_/g compared to EC and 10 mL H_2_/g compared to EC-Cr. However, this decline is not essential as the production reached approximately 60 mlH_2_/g, which is equally intriguing. It presents an opportunity to implement a complete sustainability process and maximize the biomass of E *Crassipes.* The EC-Pb biomass, which had a greater capacity for Pb adsorption, had a more significant impact on biohydrogen production than the EC-Cr biomass, which had slightly higher biohydrogen production. Therefore, it can be inferred that a higher adsorption capacity leads to a lower biohydrogen production capacity.

There are limited studies in the literature regarding the impact of heavy metals on biohydrogen production. However, previous research [12,13,14] conducted bioethanol production under similar conditions, which resulted in a 25% decrease in bioethanol production from heavy metal-loaded biomass. Table 5 presents a synopsis of studies on biohydrogen production. All these investigations were the productions of biohydrogen through dark fermentations.

The authors of [44] utilized the *E. crassipes* biomass to produce biohydrogen, yielding 73 H_2_/g. Similarly, ref. [48] investigated bioprocessing via the saccharification of lucerne, obtaining a yield of 55 mL H_2_/g. Variability was observed in the substrates used. In more specialized processes, such as those described in [51,52], celluloses were genetically modified, leading to improved hydrogen production. Also, where the production of hydrogen is realized with the bacteria of genera *Enterobacter*, *Bacillus*, and *Clostridium*, the generation of this biofuel is better, around 100% [53,54,55,56,57].

However, a biomass residue without heavy metals is what remains in this process of the mixture of the *E. crassipes* plant and biosolid (WTRs) and has potential as a biofertilizer due to its physicochemical characteristics. The anaerobic fermentation of organic matter produces an organic residue with exceptional fertilizing properties, comprising an average of 8.5% organic matter, 2.6% nitrogen, 1.5% phosphorus, 1.0% potassium, and a pH of 7.5 [58,59,60,61,62,63,64]. The material used with heavy metals must finally be disposed of as hazardous waste.

## 4. Conclusions

The biomass of *E. crassipes* has a considerable quantity of cellulose and hemicellulose, which makes it a good source of raw material for bioremediation and producing biofuels such as hydrogen. An economical and easy-to-implement method for the treatment of wastewater contaminated with heavy metals was designed and evaluated, producing a bioremediation process with dry and ground *E. crassipes* biomass, treating two types of water contaminated with Cr (VI) and Pb (II), in which around 85% was decontaminated by treating 800 mL and 90% and treating 1400 mL, respectively, with an initial concentration of 1000 mg/L.

The biomass used in this bioremediation process achieved the next level of sustainability due to its great molecular power to adsorb heavy metals where three biohydrogen production systems were designed.

This study investigated the biological production of hydrogen through batch fermentation, using biomass loaded with heavy metals as a source. The presence of Cr (IV) in *E. crassipes* resulted in a 14% decrease in biohydrogen yield, while the presence of Pb (II) led to a 26% decrease. Notably, the biomass of *E. crassipes* without contaminants produced a higher yield of hydrogen (81.7 mL H_2_/g glucose).

However, if it is affected by the presence of heavy metal biomass, it was not significant; we concluded that a large-scale environmental sustainability process can be implemented to remediate water contaminated with heavy metals and subsequently produce biohydrogen. The findings indicate that the *E. crassipes* biomass can be utilized in various sustainable processes. These processes can be combined to create a megaproject that benefits the environment. This technology is based on nature and can decontaminate industrial wastewater while also generating biofuel for the future, such as hydrogen.

## Figures and Tables

**Table 1:** Adsorption models.

Model Thomas	$\ln\frac{Co}{C}-1=\frac{Kthqm}{Q}-KthCoTb$ (1)
Model Carreño	q = $\frac{QTbCo}{M}-\frac{QTbC}{M}-\frac{\varepsilon VCo}{M}$ (2)

where Co: initial concentration; C: final concentration; V: volume; Kth: Thomas constant (ml/mg*min); q: adsorption capacity (mg/g biomass); m: biomass (g); Q: flow rate (ml/min); Tb: time of rupture (min).

**Table 2:** Characterizations of biomass of *E. crassipes*.

Lignin (%)	Cellulose (%)	Hemicellulose (%)	Other (%)	Reference
9	23	43	23	Present
1.1	17.3	24.7		[39]
4.1	19.7	27.1		[40]
1.1	17.3	24.7		[44]
11	31	27	10	[45]
11	27	27	10	[46]
12	36	42		[47]

**Table 3:** Physicochemically characterized EC-Cr sample.

Element	Weight (g)	Percentage %
Carbon	43.64	44.67
Oxygen	45.15	39.94
Cr (VI)	12.13	14.37

**Table 4:** Physicochemically characterized EC-Pb sample.

Element	Weight (g)	Percentage %
Carbon	43.64	43.67
Oxygen	45.15	38.94
Pb (II)	15.6	16.6

**Table 5:** Resumed process of biohydrogen productions.

	Biomass	Yield mL H_2_/g
Present research	*Crassipes*	81.7
Present research	*Crassipes*-Cr (VI)	71
Present research	*Crassipes*-Pb (II)	60
[44]	*Crassipes*	73
[48]	Alfalfa	55.6
[49]	waste peach pulp	59
[50]	Alternanthera hiloxeroides.	89
[51]	cellulose	102.6
[52]	Cellulomonas biazotea	105.5
[53]	lignocellulosic biomass	108
[54]	Performance of clostridium species	120
[55]	*Enterobacter*, *Bacillus*, and *Clostridium*	109
[56]	Performance of clostridium species *Bacillus*	110
[57]	Performance of clostridium species	112

## Data Availability

The datasets used and/or analyzed during the current study are available from the corresponding author upon reasonable request.
